# Predictors of sedation period for critical illness patients focusing on early rehabilitation on the bed

**DOI:** 10.1038/s41598-022-18311-8

**Published:** 2022-08-18

**Authors:** Yosuke Morimoto, Tsubasa Watanabe, Masato Oikawa, Masatoshi Hanada, Motohiro Sekino, Tetsuya Hara, Ryo Kozu

**Affiliations:** 1 Department of Physical Therapy, Faculty of Rehabilitation, Kobe Gakuin University, 518 Ikawadanicho, Arise, Nishi-ku, Kobe, Hyogo 651-2180 Japan; 2 Department of Rehabilitation Medicine, Nagasaki University Hospital, Nagasaki, Japan; 3 Department of Cardiopulmonary Rehabilitation Science, Nagasaki University Graduate School of Biomedical Sciences, Nagasaki, Japan; 4 Division of Intensive Care, Nagasaki University Hospital, Nagasaki, Japan; 5 Department of Anesthesiology, Nagasaki University Graduate School of Biomedical Science, Nagasaki, Japan

**Keywords:** Medical research, Risk factors

## Abstract

There are various interventions of rehabilitation on the bed, but these are time-consuming and cannot be performed for all patients. The purpose of this study was to identify the patients who require early mobilization based on the level of sedation. We retrospectively evaluated the data of patients who underwent physical therapy, ICU admission of > 48 h, and were discharged alive. Sedation was defined as using sedative drugs and a Richmond Agitation–Sedation Scale score of < − 2. Multiple regression analysis was performed using sedation period as the objective variable, and receiver operating characteristic (ROC) curve and Spearman’s rank correlation coefficient were performed. Of 462 patients admitted to the ICU, the data of 138 patients were analyzed. The Sequential Organ Failure Assessment (SOFA) score and non-surgery and emergency surgery cases were extracted as significant factors. The ROC curve with a positive sedation period of more than 3 days revealed the SOFA cutoff score was 10. A significant positive correlation was found between sedation period and the initial day on early mobilization. High SOFA scores, non-surgery and emergency surgery cases may be indicators of early mobilization on the bed in the ICU.

## Introduction

Although the survival rate of patients with critical illness has been improved by medical advances, the survivors frequently suffer from long-term problems^1,2^. In 2012, Needam et al.^3^ advocated post-intensive care syndrome (PICS) that includes new or worsening problems in physical, cognitive, or mental health status arising after a critical illness. The primary outcomes in intensive care are not only survival rates but also long-term problems such as PICS.

In patients with PICS, an important task in rehabilitation is prevention or inhibition of intensive care unit (ICU)-acquired weakness (ICU-AW) that induces skeletal muscle dysfunction^4^. Five major risk factors, and their related potential measures to prevent ICU-AW, can be identified, including multiple organ failure, immobility, hyperglycemia, and use of corticosteroids and neuromuscular blockers^5^. In the Clinical Practice Guidelines for the Prevention and Management of Pain, Agitation/Sedation, Delirium, Immobility, and Sleep Disruption in Adult Patients in the ICU, immobility has been added as a new problem in patients with critical illness^6^. It has been recognized that early mobilization is a general and important treatment strategy in the ICU.

Regarding the effectiveness of early mobilization, previous studies have reported about earlier extubation^7^, reduced length of stay (LOS) in the ICU and hospital^8^, and improvement of delirium^9^, physical functions^10^, activities of daily living (ADL), health-related quality of life at discharge^7,11^, and mortality^12^. Moreover, Moon et al.^13^ reported that early mobilization was feasible and safe.

Prolonged sedation causes bed rest, which is closely related to immobility. Harrold et al.^14^ reported that sedation was the most common barrier to mobilization. In recent years, several studies have reported about in-bed exercises, such as cycling^15^, neuromuscular stimulation^16^, and passive tilting^17^. However, no evidence has still been established to support these approaches on the bed.

Mobilization using such devices requires purchase costs and massive effort^18^. Considering the limitations of medical and nursing staff workload, it is not practical to apply early mobilization on the bed for all critically ill patients who are sedated^19^. In contrast, long-term problems, such as ICU-AW for ICU survivors, should not be disregarded, and they should be subjected to early mobilization that may be effective even in the case of insufficient evidence and taking time and effort. The purpose of this study was to identify the patients who require early mobilization based on the level of sedation.

## Methods

### Study design and subjects

This investigation was designed as a single-center, retrospective cohort study. Subjects were survivors who were admitted to Nagasaki University Hospital from April 2016 to March 2017 and stayed in the ICU which managed by intensivists. The inclusion criteria were staying in the ICU for > 48 h, aged ≥ 18 years, and underwent physical therapy. We excluded patients with an inability of gait before admission to the ICU, complications of neuromuscular disease and amputation, implantation of a left ventricular assist device, lung transplantation, and treated with extracorporeal membrane oxygenation and intra-aortic balloon pump.

### Measurements

The baseline characteristics of patients before ICU admission pertaining to age, sex, height, weight, body mass index, diagnosis, Charlson Comorbidity Index (CCI), and continuous renal replacement therapy (CRRT) were collected from medical records, and subjects’ ADL were evaluated using The Eastern Cooperative Oncology Group Performance Status (ECOG-PS) and Katz index. Although ECOG-PS is generally used for cancer patients, its usefulness as a prognostic value for general critically ill patients at preadmission has also been reported^20^. At ICU admission, the Sequential Organ Failure Assessment (SOFA) score, catecholamine index (dopamine + dobutamine + (epinephrine + norepinephrine) × 100 μg/kg/min), was evaluated. During ICU stay, details regarding complication of sepsis, treatment of CRRT and mechanical ventilation, presence of surgery and enteral nutrition, blood parameters (albumin, lactate, and C-reactive protein), sedation period, and LOS in the ICU were collected from medical records. At discharge from the hospital, ECOG-PS, Katz index, and LOS in the hospital were collected from medical records.

### Evaluation tool of ADL

The Eastern Cooperative Oncology Group Performance Status scoring was defined as follows: 0, fully active, able to perform all pre-disease activities without restriction; 1, restricted in physically strenuous activity but ambulatory and able to perform work of a light or sedentary nature; 2, ambulatory and capable of all self-care but unable to perform any work activities, as well as up for approximately > 50% of waking hours; 3, capable of only limited self-care, confined to bed or chair for > 50% of waking hours; and 4, completely disabled, cannot perform any self-care, totally confined to bed or chair^21^. The Katz index ranks the adequacy of performance in the six functions of bathing, dressing, toileting, transferring, continence, and feeding. Patients are scored as yes/no for independence in each of the six functions. A score of 6 indicates full function, 4 indicates moderate impairment, and ≤ 2 indicates severe functional impairment^22^.

### Sedation

The sedation period was defined as the number of days on which the Richmond Agitation–Sedation Scale score was < − 2 with the administration of propofol, dexmedetomidine hydrochloride, or midazolam at midnight. The prolonged sedation period was defined as the days of sedation period of ≥ 3 days. In the clinical setting, there is no awakening protocol because of ICU which managed by intensivists, and each intensivist adjusts the sedative medicine and judges extubation according to careful observation of the patient’s condition^23^.

### Early mobilization

The mobilization was performed according to a previously reported protocol^24^. We defined early mobilization as the level of sitting on the edge of the bed or higher, as applicable to 3 in ICU mobility scale (IMS), within the first 2 days during the patients' ICU stay^25^. Details regarding the initial day of intervention, sitting on the edge of the bed (IMS = 3), standing (IMS = 4), and gait (IMS ≥ 7) were collected from medical records.

### Statistical analysis

A regression analysis was performed to identify the clinical variables associated with sedation period as a continuous variable. Candidate factors were selected based on a literature review, clinical expertise, and those that significantly contributed to the outcome of the single linear regression analysis (p < 0.05). In multiple linear regression models, candidate factors were selected according to a literature review, clinical expertise, and their significant contributions to the outcome of the simple linear regression analysis. The sample size was based on the report by Peter et al., and the number of factors suitable for multivariate analysis was extracted^26^. These models were applied to evaluate whether these variables were the predictors of sedation period for subjects. In addition, we performed three subanalyses. First, the SOFA cutoff score for determining sedation period for ≥ 3 days was evaluated using a receiver operating characteristic (ROC) curve. The highest value of Youden’s Index was used as the criterion for determining the cutoff point. Second, Spearman’s rank correlation coefficient was calculated to determine whether the sedation period correlated with the progression of early mobilization. Third, the Wilcoxon rank-sum test was used to examine differences in sedation period due to changes in ADL as changes in ECOG-PS and Katz index at hospital discharge compared with baseline. There was no subject who showed improved ADL. We divided the subjects into two groups, the preservation group that maintained the score of ECOG-PS and Katz index at hospital discharge compared with baseline and the decrease group that decreased the score of that. All analyses were conducted using the JMP^®^ pro 13 software, version 13.0.0 (SAS Institute Inc., Cary, NC, USA). Data are expressed as median (25th to 75th percentile), or percentage, as appropriate. The level of significance was 0.05 for all tests.

### Ethics declarations

This study was approved by the Human Ethics Review Committee of Nagasaki University Hospital (reference number 19070826). The informed consent for data analysis and publication in this study was waved by the Human Ethics Review Committee of Nagasaki University Hospital and was obtained in the form of opt-out on the web-site. Those who rejected were excluded. This study was conducted in accordance with the Declaration of Helsinki.

### Consent for publication

The research presents no more than minimal risk of harm to subjects and involves no procedures; hence, the requirement for written consent was waived.

## Results

### Patient characteristics

**Figure 1:**
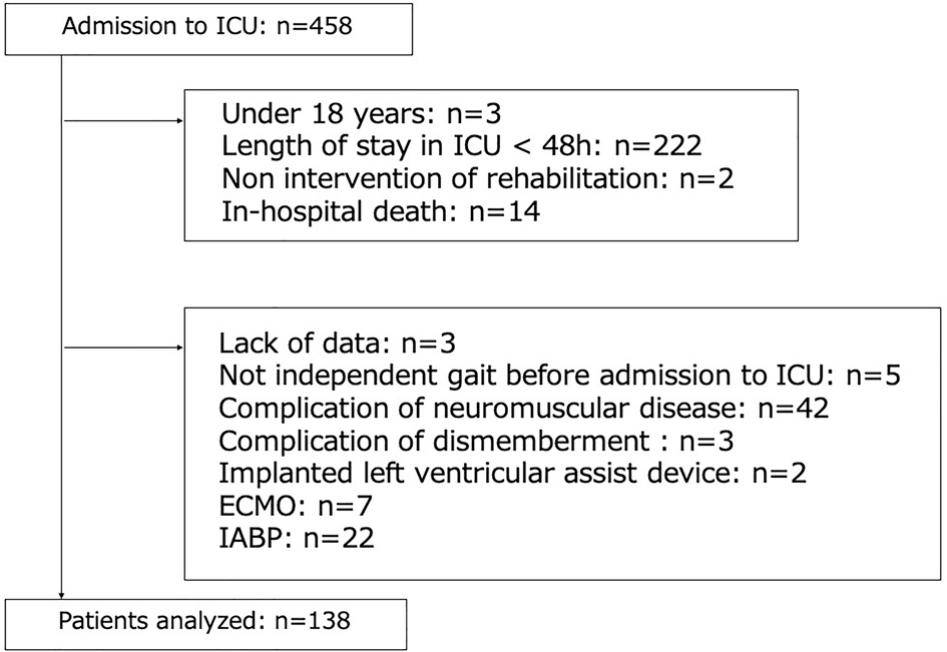
Flow diagram. *ICU* intensive care unit, *ECMO* extracorporeal membrane oxygenation, *IABP* intra-aortic balloon pump.

During the study period, 458 patients were admitted to the ICU; 241 patients did not satisfy any inclusion criteria, and 79 patients satisfied the exclusion criteria. Finally, the data of the remaining 138 patients were analyzed (Fig. 1). Table 1 shows the patient characteristics separately for total and surgical cases. Cardiovascular and digestive organ disorders accounted for > 80% of the disease, and the CCI score was low. Regarding ADL at baseline, median ECOG-PS score was 1 (range, 0–2) and median Katz index score was 6 (range, 6–6). As a supplement, Katz index score was 6 out of 6 in the majority of patients.

**Table 1:** Characteristics.

	Total (n = 138)	RASS < − 2 < 3 days (n = 79)	RASS < − 2 ≥ 3 days (n = 59)	p-value
Age, years, median [IQR]	67.5 [57.8–78]	71.0 [61.0–79.0]	62.0 [54.0–76.0]	0.01
Sex, female, n (%)	51 (37)	30 (38.0)	21 (35.6)	0.86
Height, cm, median [IQR]	161.0 [153.8–168.0]	160.0 [512.0–167.0]	163.0 [156–168]	0.20
Weight, kg, median [IQR]	57.5 [49–67]	56.0 [49.0–64.0]	61.0 [49.0–71.0]	0.06
BMI, kg/m^2^, median [IQR]	22.27 [20.06–24.86]	22.31 [19.96–24.17]	22.09 [20.76–26.37]	0.21
**Disease**				
Cardiovascular, n (%)	60 (43.5)	48 (60.8)	12 (20.3)	< 0.01
Digestive organ, n (%)	56 (40.6)	26 (32.9)	30 (50.8)	0.04
Pulmonary, n (%)	8 (5.8)	3 (3.8)	5 (8.5)	0.29
Others, n (%)	14 (10.1)	2 (2.5)	12 (20.3)	< 0.01
Complication of sepsis, n (%)	31 (22.5)	7 (8.9)	24 (40.7)	< 0.01
Charlson Comorbidity Index, median [IQR]	1 [0–2]	1 [0–3]	1 [0–2]	0.19
Case of hemodialysis, n (%)	16 (11.6)	11 (13.9)	5 (8.5)	0.42
Katz index, median [IQR]	6 [6–6]/6 [4–6]	6 [6–6]/6 [4–6]	6 [6–6]/5 [3–6]	1.00/0.02
ECOG-PS, median [IQR]	1 [0–2]/2 [1–3]	1 [0–1]/2 [1–3]	0 [0–2]/2 [1–4]	0.02/0.02
SOFA score, median [IQR]	9 [7–11]	8 [6–9]	11 [9–13]	< 0.01
**Catecholamine index, median [IQR]**	5.5 [1.6–18.6]	3.6 [1.1–10.0]	12.8 [5.0–40.0]	< 0.01
Administration of dopamine, n (%)	74 (53.6)	50 (63.3)	24 (40.7)	0.02
Dose of dopamine, μg/kg/min, median [IQR]	3.59 [2.46–5.36]	3.55 [2.46–5.29]	3.63 [2.55–5.74]	0.74
Administration of dobutamine, n (%)	7 (5.1)	5 (15.8)	2 (3.4)	0.70
Dose of dobutamine, μg/kg/min, median [IQR]	3.47 [2.29–4.28]	3.5 [2.56–3.62]	3.48 [2.75–4.21]	1.00
Administration of epinephrine, n (%)	6 (4.3)	1 ((1.3)	5 (8.5)	0.08
Dose of epinephrine, μg/kg/min, median [IQR]	0.28 [0.04–0.50]	0.02 [0.02–0.02]	0.50 [0.05–0.50]	0.12
Administration of norepinephrine, n (%)	69 (50.0)	30 (38.0)	39 (66.1)	0.03
Dose of norepinephrine, μg/kg/min, median [IQR]	0.12 [0.05–0.30]	0.10 [0.05–0.15]	0.20 [0.07–0.45]	< 0.01
Sedation period, day, median [IQR]	2 [1–4]	1 [1–2]	4 [3–6]	< 0.01
**Mechanical support**				
Mechanical ventilation, n	129 (93.5)	72 (91.1]	57 (96.6)	0.20
Ventilation time, hour, median [IQR]	35.5 [14.0–72.5]	17.0 [6.0–30.0]	83 [60–137]	< 0.01
Continuous hemodiafiltration, n (%)	98 (71.0)	12 (15.2)	28 (47.5)	< 0.01
Enteral nutrition, n (%)	48 (37.2)	23 (29.5)	25 (49.0)	0.03
Non-surgery, n (%)	30 (21.7)	6 (7.6)	24 (40.7)	< 0.01
**Blood findings**				
Albumin, g/dL, median [IQR]	2.7 [2.4–3.0]	2.7 [2.4–2.9]	2.8 [2.4–3.2]	0.24
Lactate, nmol/L, median [IQR]	1.9 [1.1–3.1]	1.7 [1.1–2.9]	2.2 [1.1–3.9]	0.06
CRP, mg/L, median [IQR]	1.9 [0.5–10.3]	0.8 [0.2–2.3]	9.1 [2.1–18.8]	< 0.01
Length of stay in ICU, day, median [IQR]	5 [4–7]	4 [4–5]	7 [5–9]	< 0.01
**Mobilization**				
First intervention, day, median [IQR]	2.0 [2.0–4.0]	2.0 [2.0–2.0]	4.0 [2.0–5.0]	< 0.01
Sitting, day, median [IQR]	3.5 [2.0–6.0]	2.0 [2.0–3.0]	6.0 [4.0–8.0]	< 0.01
Standing, day, median [IQR]	4.0 [2.0–8.0]	2.0 [2.0–3.0]	7.0 [4.8–9.3]	< 0.01
Gait, day, median [IQR]	6.0 [3.0–9.0]	5.0 [4.0–8.0]	10.0 [7.0–17.0]	< 0.01
Length of stay in hospital, median [IQR]	32.5 [24.0–56.3]	30.0 [21.0–51.0]	39.0 [28.0–63.0]	0.01
**Surgical findings**				
Case of surgery, n (%)	108 (78.3)	73 (92.4)	35 (59.3)	< 0.01
Case of emergency surgery, n (%)	46 (42.6)	21 (26.6)	25 (42.4)	< 0.01
Time of surgery, minute, median [IQR]	313 [214–519]	374 [250–571]	271 [160–471]	0.16
Time of anesthesia, minute, median [IQR]	383 [288–592]	441 [332–666]	325 [224–543]	0.10

*IQR* interquartile range, *n* number, *BMI* body mass index, *ECOG-PS* The Eastern Cooperative Oncology Group Performance Status, *SOFA* Sequential organ failure assessment, *CRP* C-reactive protein.

### At ICU admission

The median SOFA score was 9 (range, 7–11), and there were 31 patients (22.5%) with a complication of sepsis. There were more surgical cases than medical cases (n = 116, 78.3% vs. n = 22, 21.7%). Most patients required mechanical support such as mechanical ventilation (n = 129, 93.5%) and CRRT (n = 98, 71.0%). The median first mobilization day, which progressed after ICU admission, of intervention, sitting, standing, and gait was 2.0 days (range, 2.0–4.0 in overall and 2.0–3.0 in surgical cases), 3.5 days (range, 2.0–6.0 in overall and 2.0–4.8 in surgical cases), 4.0 days (range, 2.0–8.0 in overall and 2.0–6.0 in surgical cases), 6.0 days (range, 3.0–9.0) in overall and 7.0 days (range, 4.0–9.0) in surgical cases, respectively.

### Single linear regression

A single linear regression model was analyzed to investigate the predictor variables that had a significant influence on the sedation period (Tables 2, 3). The extracted significant factors, in both overall and surgical cases, were cardiovascular and digestive organ, complication of sepsis, SOFA score, catecholamine index, case of continuous hemodiafiltration, lactate and CRP. In addition to these factors, in overall, age, weight, BMI, others in disease, ECOG-PS, non-surgery cases were extracted. In surgery cases, case of emergency surgery were extracted.

**Table 2:** Association to sedation period by single and multiple liner regression.

	Single analysis			Multiple analysis		
	Estimate	95% CI	p value	Estimate	95% CI	p value
Age, per a year	− 0.05	− 0.08 to − 0.02	< 0.01	− 0.01	− 0.04 to 0.01	0.30
Sex, female	0.03	− 0.37 to 0.43	0.89			
Height, per 1 cm	0.01	− 0.03 to 0.05	0.70			
Weight, per 1 kg	0.04	0.11 to 0.07	0.01			
BMI, per 1 kg/m^2^	0.13	0.05 to 0.21	0.01	0.07	− 0.01 to 0.15	0.07
**Disease**						
Cardiovascular	− 1.42	− 2.03 to − 0.80	< 0.01	0.49	− 0.32 to 1.20	0.22
Digestive organ	0.77	0.11 to 1.42	0.02	0.52	− 0.14 to 1.18	0.11
Pulmonary	− 0.01	− 1.39 to 1.41	0.99			
Others	1.80	0.76 to 2.84	< 0.01	0.18	− 0.82 to 1.18	0.72
Complication of sepsis	0.82	0.38 to 1.26	0.01	− 0.12	− 0.68 to 0.43	0.66
Charlson Comorbidity Index, per a point	− 0.15	− 0.35 to 0.04	0.13			
Case of hemodialysis	− 0.50	− 1.10 to 0.10	0.10			
Katz index, per a point	0.19	− 0.52 to 0.91	0.59			
ECOG-PS, per a point	− 0.44	− 0.81 to − 0.06	0.02	− 0.34	− 0.73 to 0.05	0.09
SOFA score, per a point	0.26	0.15 to 0.37	< 0.01	0.13	0.01 to 0.26	0.04
Catecholamine index, per a point	0.03	0.01 to 0.05	0.01			
**Mechanical support**						
Case of mechanical ventilation	0.73	− 0.03 to 1.50	0.06			
Case of continuous hemodiafiltration	0.84	0.44 to 1.25	< 0.01	0.29	− 0.22 to 0.79	0.26
Enteral nutrition	0.13	0.32 to − 0.58	0.55			
Non-surgery cases	1.30	0.88 to 1.72	< 0.01	0.94	0.38 to 1.49	< 0.01
**Blood findings**						
Albumin, per 1 g/dL	0.47	− 0.24 to 1.17	0.19			
Lactate, per 1 nmol/L	0.24	0.03 to 0.45	0.03	0.07	− 0.12 to 0.26	0.48
CRP, per 1 mg/L	0.13	0.09 to 0.17	< 0.01	0.04	− 0.02 to 0.10	0.21

*BMI* body mass index, *ECOG-PS* The Eastern Cooperative Oncology Group Performance Status, *SOFA* sequential organ failure assessment, *CRP* C-reaction protein.

**Table 3:** Association to sedation period by single and multiple liner regression in surgery cases.

	Single analysis			Multiple analysis		
	Estimate	95% CI	p value	Estimate	95% CI	p value
Age, per a year	− 0.00	− 0.03 to 0.03	0.91			
Sex, female	0.00	− 0.35 to 0.35	0.99			
Height, per 1 cm	− 0.00	− 0.03 to 0.03	0.99			
Weight, per 1 kg	0.01	− 0.01 to 0.04	0.31			
BMI, per 1 kg/m^2^	0.06	− 0.03 to 0.14	0.19			
**Disease**						
Cardiovascular	− 0.49	− 0.81 to − 0.17	< 0.01	− 0.29	− 1.34 to 0.76	0.47
Digestive organ	0.45	0.12 to 0.77	< 0.01	0.02	− 1.06 to 1.09	0.97
Others	0.38	− 0.63 to 1.39	0.48			
Complication of sepsis	0.74	0.32 to 1.17	< 0.01	− 0.56	− 1.29 to 0.18	0.13
Charlson Comorbidity Index, per a point	− 0.12	− 0.30 to 0.06	0.19			
Case of hemodialysis	− 0.11	− 0.59 to 0.37	0.65			
Katz index, per a point	0.02	− 0.61 to 0.66	0.94			
ECOG-PS, per a point	− 0.30	− 0.64 to 0.04	0.08			
SOFA score, per a point	0.25	0.15 to 0.34	< 0.01	0.13	0.01 to 0.25	0.04
Catecholamine index, per a point	0.05	0.03 to 0.07	< 0.01	0.02	− 0.01 to 0.06	0.14
**Mechanical support**						
Case of mechanical ventilation	0.24	− 0.55 to 1.03	0.55			
Case of continuous hemodiafiltration	0.96	0.60 to 1.31	< 0.01	0.44	− 0.01 to 0.89	0.05
Case of enteral nutrition	− 0.12	− 0.51 to 0.28	0.56			
**Blood findings**						
Albumin, per 1 g/dL	0.13	− 0.53 to 0.80	0.69			
Lactate, per 1 nmol/L	0.33	0.13 to 0.53	< 0.01	− 0.34	− 0.85 to 0.16	0.18
CRP, per 1 mg/L	0.03	0.05 to 0.16	< 0.01	− 0.03	− 0.07 to 0.02	0.29
**Surgical findings**						
Case of emergency surgery	0.70	0.39 to 1.01	< 0.01	0.59	0.19 to 1.00	< 0.01
Time of surgery, per a minute	− 0.00	− 0.00 to 0.00	0.93			
Time of anesthesia, per a minute	− 0.00	− 0.00 to 0.00	0.82			

*BMI* body mass index, *ECOG-PS* the Eastern Cooperative Oncology Group Performance Status, *SOFA* sequential organ failure assessment, *CRP* C-reaction protein.

### Correlation of mobilization and sedation period

**Figure 2:**
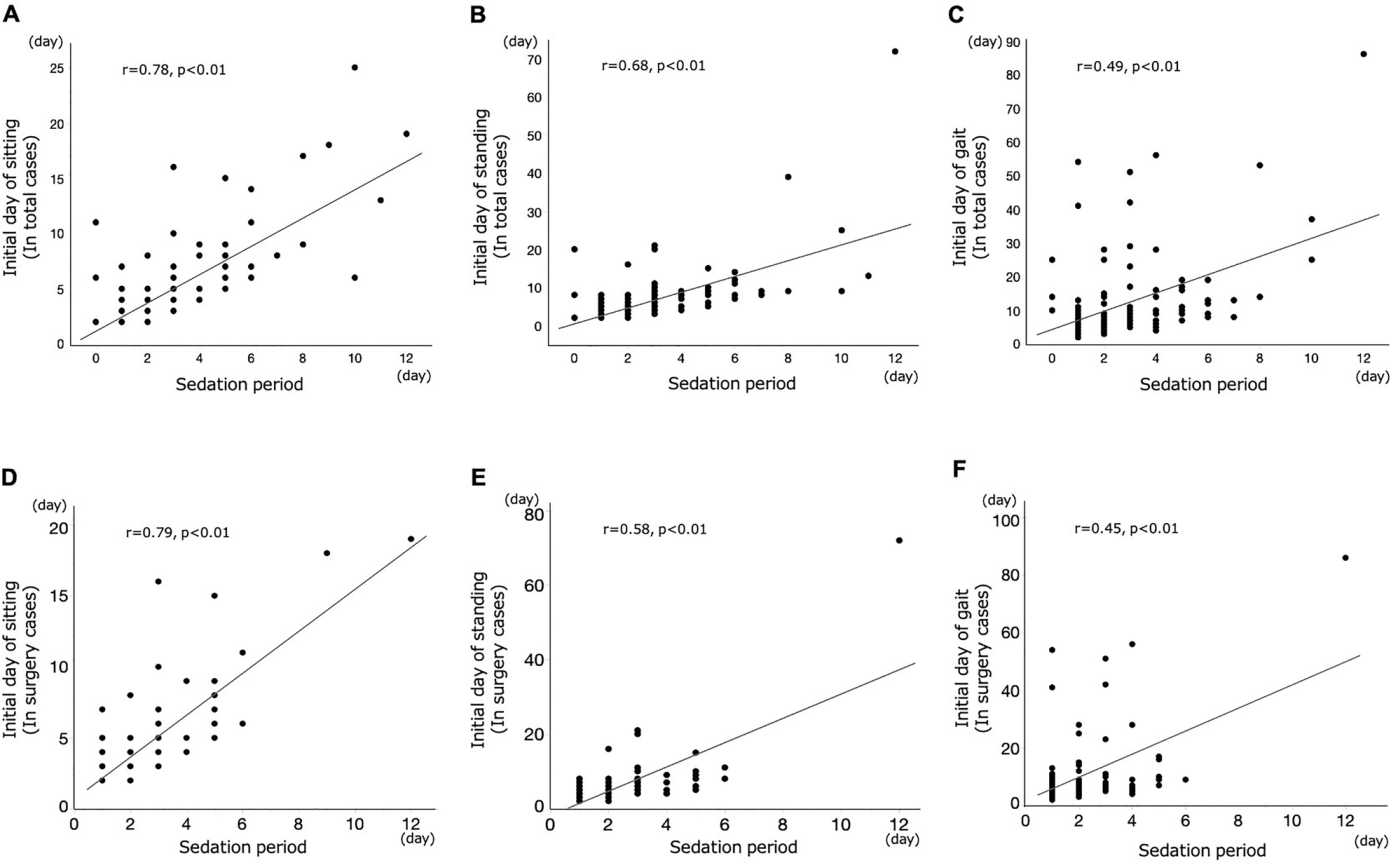
Correlation between mobilization and sedation (**A**–**C**) in overall and (**D**–**F**) in surgery cases. (**A**,**D**) Between initial day of sitting on the edge of the bed and sedation period. (**B**,**E**) Between initial day of standing and sedation period. (**C**,**F**) Between initial day of gait and sedation period.

The differences between each first day of mobilization and sedation period are depicted in Fig. 2. Positive correlations in both total and surgical cases were found between the sedation and initial day of sitting (*r* = 0.78; p < 0.01 and *r* = 0.79; p < 0.01), standing (*r* = 0.68; p < 0.01 and *r* = 0.58; p < 0.01), gait (*r* = 0.49; p < 0.01 and *r* = 0.45; p < 0.01).

### Differences between change in ADL and sedation period

**Figure 3:**
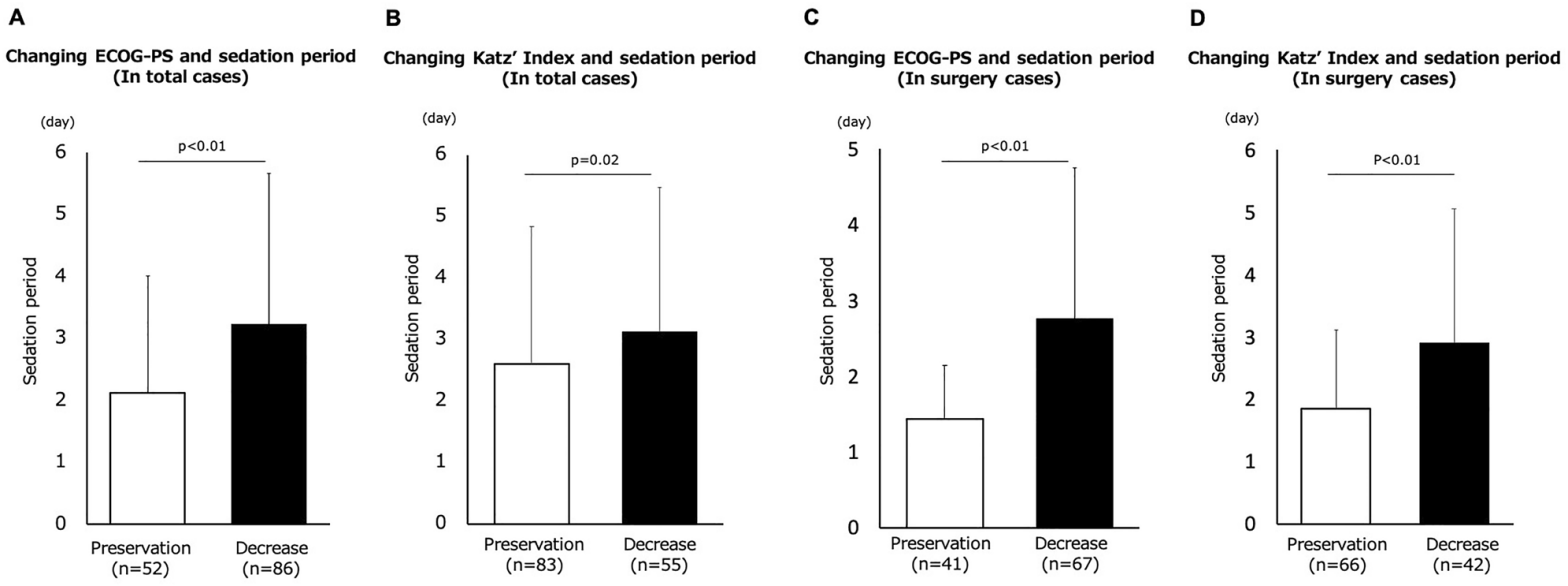
Comparison of the sedation period between two groups classified by changing score of ECOG-PS (**A**,**C**) and Katz index (**B**,**D**), respectively, in overall (**A**,**B**) and in surgery cases (**C**,**D**).

We evaluated ADL using ECOG-PS and Katz index at baseline and hospital discharge. Patients were divided into two groups, preservation and reduction, and the changes in ECOG-PS and Katz index were compared at two time points. In overall, there were 52 patients (37.7%) in terms of ECOG-PS and 83 patients (60.1%) in terms of Katz index in the preservation group and 86 patients (62.3%) in terms of ECOG-PS and 55 patients (39.9%) in terms of Katz index in the reduction group. In surgical cases, there were 41 patients (38.0%) in terms of ECOG-PS and 66 patients (61.1%) in terms of Katz index in the preservation group and 67 patients (62.0%) in terms of ECOG-PS and 55 patients (38.9%) in terms of Katz index in the reduction group. There was no patient who showed improved ADL at hospital discharge compared with baseline. In both evaluations, the sedation period in the preservation group was significantly longer than that in the reduction group (Fig. 3).

### Multiple linear regression

In overall, there was a 0.14-day increase in sedation period for each positive step on the SOFA score (p = 0.04) and the sedation period in non-surgery cases, was increased by 0.94 days (p < 0.01). In surgical cases, there was a 0.13-day increase in sedation period for each positive step on the SOFA score (p = 0.04) and the sedation period in case of emergency surgery was increased by 0.59 days (p < 0.01).

### ROC curve

**Figure 4:**
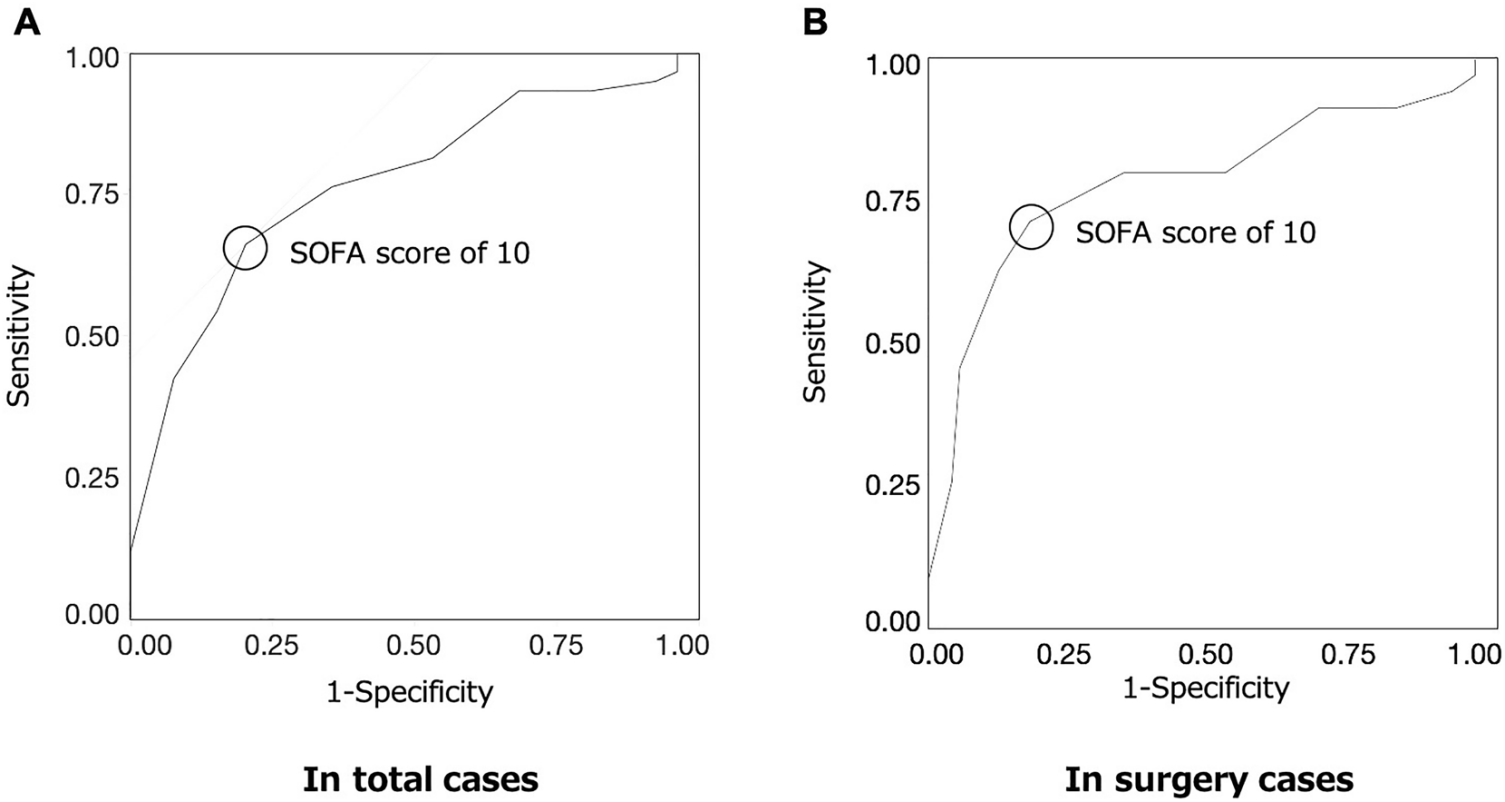
Receiver operating characteristic curve analysis for sedation period (**A**) in overall and (**B**) in surgery cases. Positive was defined as a sedation period of more than 3 days. The cutoff point of SOFA score was 10 in both: sensitivity, 0.66; specificity, 0.80; area under the curve, 0.77; standard error, 0.05 in overall and sensitivity, 0.71; specificity, 0.82; area under the curve, 0.79; standard error, 0.05 in surgery cases.

The ROC curve for determining the sedation period that prolonged for ≥ 3 days is shown in Fig. 4. The area under the curve for the SOFA score was 0.77 in overall and 0.79 in surgical cases. The cutoff value for the SOFA score for determining the sedation period of ≥ 3 days was 10 (sensitivity, 0.66; specificity, 0.80 in overall and sensitivity, 0.71; specificity, 0.82 in surgical cases).

## Discussion

The major findings of this study include: (1) a high SOFA score and non-surgery cases were significantly associated with prolonged sedation period, and a SOFA score of 10 was expected to prolong the sedation period for ≥ 3 days, and (2) delayed mobilization getting out of bed and decrease in ADL were related to prolonged sedation period.

Overall, severe critically ill patients with non-surgery should start mobilization on the bed as rapidly as possible because they may experience delayed mobilization and decrease in ADL after hospital discharge. Then, in surgical cases, the case of emergency surgery should start mobilization like in overall. Although the SOFA score is a simple, effective method to describe organ dysfunction/failure in critically ill patients^27^, Arts et al.^28^ reported that the reliability and accuracy of SOFA scoring among physicians are better. Evaluation using the SOFA score is performed in almost Japanese ICUs, because the calculation of SOFA score is one of the requirements for medical fees related to ICU management in Japan. Although the ABCDE bundle is implemented in an institution, it is inevitable to prolong the sedation period in more severe patients having high SOFA scores. Furthermore, Peeters et al.^29^ reported that the severity of illness, expressed as SOFA score, particularly influenced the pharmacodynamics, and to a minor degree, the pharmacokinetics, and deeper levels of sedation were found with increasing SOFA scores. According to Ad Hoc Committee for Early Rehabilitation by the Japanese Society of Intensive Care Medicine, early mobilization is defined to start mobilization within 2 days of admitting the ICU^30^. We defined that positive in ROC curve was the sedation for more than 3 days, because mobilization from the third day after admitting ICU is not considered to be early mobilization. This cutoff score may help physiotherapy staff in deciding whether to start early mobilization on the bed.

It is well known that early mobilization leads to better functional outcomes at hospital discharge^7^. In this study, a delay in mobilization was found to correlate with prolonged sedation period. As represented by the ABCDE bundle, it is common to avoid deep sedation as much as possible in recent intensive care, and the same strategy was applied in this study^31^. Watanabe et al.^32^ reported that in terms of the rehabilitation activities performed in the ICU, both in-bed exercises and the total duration of rehabilitation activity were significantly shorter in the ICU-AW group than in the non-ICU-AW group. Although mobilization getting out of the bed should be started as early as possible, mobilization on the bed may need to be started with reference to the two predictors demonstrated in this study, even during the difficult sedation period. Considering that a decrease in ADL at hospital discharge was significantly associated with prolonged sedation period, the ability to start mobilization on the bed may be important in improving outcome in patients with critical illness. To achieve these, the analgesia-sedation protocols in the ICU should be focused on using minimal or no sedation and using preemptive analgesia before mobilization according to the ABCDE bundle^31^.

There were several limitations in this study. First, it was a single-center study. How to awake depends on the characteristics of the hospital, such as according to the routine awakening protocol. The results of this study may not be helpful if skilled intensivists do not awaken patients or multiple occupations practice the ABCDE bundle. Second, this was a retrospective study. Although we conducted multivariate analysis, we cannot rule out the possibility that there are other significant factors in addition to those extracted in this study, because we analyzed only existing medical records. Further research using a prospective study and considering other factors as well is required for a detailed evaluation. Third, there was no system to confirm that it was fully compliant about mobilization protocol. Not a little, the content of mobilization may have been changed at the discretion of the individual physiotherapy staff. Forth, patients with lung transplant surgery were not included in this study because usual early rehabilitation protocol was not used in them. Fifth, since this study does not calculate the sample size as an exploratory study, the statistical power of the sample is unknown.

## Conclusions

Multiple analyses were performed to obtain an index of the type of patient early mobilization that should be performed on the bed. We recommended that patients who SOFA score of ≥ 10, non-surgical and emergency surgery patients should be identified as requiring prolonged sedation and poor functional outcome/prognosis and should be started mobilization as soon as possible. These results may help promote early mobilization on the bed for patients with critical illness in the ICU even in the case of insufficient evidence and taking time and effort.

## Data Availability

The datasets generated and analysed during the current study are not publicly available due to data protection but are available from the corresponding author on reasonable request.
